# The Application of Ultrasonography in Risk Assessment of Reflux and Aspiration in Diabetes Patients Undergoing Gastroparesis Surgery: Application of Ultrasonography in Diabetes Patients Undergoing Gastroparesis Surgery

**DOI:** 10.31661/gmj.v14i.4001

**Published:** 2025-10-20

**Authors:** Xiuqing Xu, Hong Bi, Jiuming Dai, Pei Gao

**Affiliations:** ^1^ Department of Anesthesiology, Binhai County People’s Hospital, China; ^2^ Department of Orthopedics, Binhai County People’s Hospital, China

**Keywords:** Diabetic Gastroparesis, Gastric Emptying, Reflux, Aspiration, Gastric Ultrasound

## Abstract

**Background:**

Diabetic gastroparesis increases the risk of reflux and macro-aspiration during surgery. This study evaluated the predictive role of gastric antral ultrasound in identifying these events in diabetic gastroparesis patients undergoing surgical procedures.

**Materials and Methods:**

A cross-sectional study was conducted on 60 diabetic gastroparesis patients at Binhai County People’s Hospital (June 2023–June 2025). Gastric antral ultrasound was performed using a SonoSite M-Turbo system, measuring cross-sectional area (CSA), gastric volume (GV), and GV-to-weight ratio (GV/W). Reflux was defined as pharyngeal pH 4, and macro-aspiration was confirmed via bronchial assessment. Statistical analysis (SPSS 27.0) included independent t-tests, chi-square tests, and logistic regression.

**Results:**

Among 60 patients (34 male, 26 female; mean age 48.19 ± 7.23 years), 9 (15%) experienced reflux (n=6) or macro-aspiration (n=3). Patients with events had significantly higher CSA (3.28 ± 0.35 vs. 3.07 ± 0.29 cm², P=0.038), GV (12.15 ± 3.28 vs. 10.12 ± 2.76 mL, P=0.013), and GV/W (0.17 ± 0.05 vs. 0.15 ± 0.04 mL/kg, P=0.022). Postoperative SpO2 was lower (94.89 ± 2.11% vs. 97.45 ± 1.88%, P0.001), and gastric protease levels were higher (62.78 ± 10.92 vs. 55.88 ± 11.34 ng/mL, P=0.006) in affected patients. Logistic regression confirmed CSA (OR=1.45, 95% CI: 1.02–2.06, P=0.039), GV (OR=1.22, 95% CI: 1.04–1.43, P=0.015), and GV/W (OR=1.38, 95% CI: 1.03–1.85, P=0.031) as significant predictors.

**Conclusion:**

Gastric antral ultrasound effectively predicts reflux and macro-aspiration risk in diabetic gastroparesis patients, with elevated CSA, GV, and GV/W serving as key indicators. These findings support preoperative ultrasound screening to mitigate perioperative complications.

## Introduction

With the increasing prevalence of diabetes worldwide, diabetes related complications have become a major public health burden. Among them, diabetic gastroparesis (DGP) is a common gastrointestinal problem in diabetic patients, which can lead to abnormal gastric function under the influence of continuous high-level blood glucose, mainly manifested as delayed gastric emptying, belching, abdominal distention, nausea, vomiting, anorexia and other symptoms, especially after meals [1]. DGP will affect drug absorption to a certain extent, hinder normal eating, and then prolong the course of disease and endanger the quality of life. It is reported that about 5-12% of diabetic patients are accompanied by gastroparesis [2]. Its core pathological characteristics are gastric antral motility decline, gastric electrical rhythm disturbance and gastric pyloric duodenal coordination disorder, resulting in significantly delayed emptying of gastric contents. In this pathological state, when patients face surgical operation, the risk of perioperative reflux aspiration will rise sharply. Once the contents of the stomach reflux to the throat and aspiration into the lower respiratory tract, it can induce pneumonia, acute respiratory distress syndrome and other serious diseases [3][4].

In current clinical practice, there are still great limitations in the evaluation of gastric emptying status and aspiration risk of surgical patients.

Traditional qualitative evaluation methods are subjective and lack of objective quantitative indicators. While nuclide gastric emptying imaging, the recognized "gold standard" for the assessment of gastric emptying function, is highly accurate, it is difficult to implement routinely before surgery due to its expensive equipment, complex operation and radiation exposure risk. As a new noninvasive, real-time and reproducible bedside tool, ultrasonic cross-sectional area (CSA) measurement of gastric antrum shows good potential in the evaluation of gastric contents in healthy people and some patient groups (such as diabetes mellitus, cesarean section, obesity) [5][6]. However, its value in accurately predicting the risk of reflux aspiration in DGP, a special high-risk group, remains to be further clarified.

It should be noted that the gastric emptying disorder in patients with DGP is not homogeneous, which often manifests as the asynchrony of liquid and solid emptying (the liquid may be relatively normal, while the solid is significantly delayed), and is dynamically affected by blood glucose fluctuations, the degree of neuropathy, drugs and other factors. Existing studies mostly focus on using ultrasound to predict "whether the stomach is full" [7] or "delayed emptying" [8], but the relationship between "gastric content status" and "actual reflux aspiration" is not simple linear. The occurrence of aspiration is also highly dependent on many factors, such as the function of the lower esophageal sphincter, airway protective reflex, anesthesia operation and so on [9].

At present, there is a lack of high-quality research to clearly establish the quantitative relationship and predictive threshold between specific ultrasound parameters (such as gastric antrum CSA, gastric content volume, distribution pattern, peristaltic wave frequency / amplitude, etc.) and perioperative actual reflux aspiration events in patients with DGP. In addition, the existing guidelines are often "one size fits all" for the preoperative fasting recommendations of DGP patients, lacking individualized risk stratification strategies based on objective gastric function assessment. It is urgent to explore whether quantitative indicators based on gastric ultrasound can build an effective prediction model or risk scoring system to accurately identify "extremely high-risk reflux aspiration individuals" in DGP patients, so as to guide individualized anesthesia programs (such as selecting awake intubation, adjusting induction drugs, extending fasting time, and even delaying surgery).

In this context, this study intends to explore the application value of gastric ultrasonography in evaluating the status of gastric contents and predicting the risk of perioperative reflux aspiration in patients with diabetic gastroparesis surgery, in order to provide a basis for the development of evidence-based perioperative management plan for patients with DGP.

## Materials and Methods

### Study Design

This cross-sectional study evaluated 60 diabetic gastroparesis patients undergoing surgery at Binhai County People’s Hospital between June 2023 and June 2025 to assess the predictive role of gastric antral ultrasound in identifying reflux and macro-aspiration events during procedures. All patients were analyzed as a single cohort, with outcomes compared between those with and without reflux/macro-aspiration events.

### Eligbility Criteria

Eligible participants met the diagnostic criteria for diabetic gastroparesis as defined in Practical Diabetology (Third Edition), including: (1) a history of diabetes exceeding 5 years; (2) symptoms such as middle and upper abdominal discomfort, indigestion, anorexia, fullness, repeated hiccups, nausea, vomiting, acid regurgitation, belching, or constipation persisting for over 2 months; (3) exclusion of mechanical obstruction; and (4) presence of food residues in gastric juice after a 12-hour morning fast. All participants provided voluntary informed consent.

Patients were excluded if they had: (1) coma due to craniocerebral injury, active gastric ulcer, upper gastrointestinal bleeding, prior esophageal or gastric surgery, inability to assume the required position for ultrasound examination, or refusal to undergo ultrasound; (2) acute diabetic complications within the previous 6 months; (3) pregnancy or lactation; or (4) severe liver, kidney, hematological, peptic ulcer, cardiopulmonary, mental, or malignant diseases. Patients unable to complete required examinations or adhere to treatment protocols were also excluded.

Participants were withdrawn if they: (1) withdrew for any reason or (2) voluntarily discontinued participation.

### Procedures

All patients received hypoglycemic treatment, including blood sugar control, exercise, weight management, smoking cessation, alcohol limitation, and dietary control. Routine preoperative protocols included fasting from solid food for 8 to 12 hours (12 to 24 hours for severe cases), clear fluid fasting for at least 6 hours (reduced to 2 to 3 hours for mild cases based on individualized assessment), and preoperative administration of metoclopramide (10 mg, three to four times daily) or domperidone (10 mg, three to four times daily). Additionally, 40 mg of omeprazole was administered orally the night before and the morning after surgery.

Gastric antral ultrasound was performed using a SonoSite M-Turbo ultrasound system in abdominal imaging mode with a 2 to 5 MHz probe. Two qualified anesthesiologists, trained in gastric ultrasound, conducted bedside assessments with patients positioned in the right lateral decubitus position. The gastric antrum was imaged in the mid-sagittal plane below the xiphoid process, along the deep plane from the left liver lobe to the posterior pancreatic region. The head-tail diameter (D1) and anterior-posterior diameter (D2) of the gastric antrum were measured, and the cross-sectional area (CSA) was calculated using the formula CSA = D1 × D2 × π/4. A CSA greater than 3.40 cm² [10], indicating gastric contents exceeding 0.8 mL/kg, led to immediate suspension of surgery due to high reflux aspiration risk. If preoperative fasting violations were confirmed, surgery was terminated, and a re-examination was required before rescheduling. All surgeries were performed by the same physician. An endoscopic standard pyloroplasty surgery was performed for all patients [11].

### Study Outcomes

Gastric Emptying Indices: Gastric antral ultrasound measured the head-tail diameter (D1) and anterior-posterior diameter (D2) to calculate the CSA (cm²), gastric volume (GV, mL), and gastric volume-to-weight ratio (GV/W, mL/kg). The GV was calculated using the formula GV = 27.0 + 14.6 × CSA − 1.28 × age (years) [12]. The GV/W ratio assessed reflux aspiration risk, with thresholds: >1.5 mL/kg (high risk), 0.8 to 1.5 mL/kg (low risk), and <0.8 mL/kg (very low risk) [13].

Preoperative and Postoperative Measurements: These included mean arterial pressure (MAP, mmHg), blood oxygen saturation (SpO2, %), pharyngeal pH, and gastric protease levels (ng/mL). A pharyngeal pH >4 indicated reflux events, with a reference range for gastric protease levels of 0 to 55.39 ng/mL.

Incidence: Reflux (pharyngeal) and macro-aspiration (bronchial) events were recorded. Patients were categorized based on the presence (n=9) or absence (n=51) of reflux/macro-aspiration events.

### Statistical Methods

Data analysis was performed using SPSS 27. 0. Normally distributed measurement data were expressed as mean ± standard deviation and analyzed using independent-sample t-tests to compare patients with and without reflux/macro-aspiration events. Categorical data were analyzed using chi-square tests. A p-value <0.05 was considered statistically significant. Logistic regression was used to assess the predictive role of ultrasound-derived CSA, GV, and GV/W for reflux/macro-aspiration events.

## Results

**Table:** Table1. Comparison of Clinical Parameters Between Patients With and Without Reflux/Macro-aspiration Events (Mean ± SD)

**Parameter**	**With Events (n=9)**	**Without Events (n=51)**	**p-value**
**CSA (cm²)**	3.28 ± 0.35	3.07 ± 0.29	0.038
**GV (mL)**	12.15 ± 3.28	10.12 ± 2.76	0.013
**GV/W (mL/kg)**	0.17 ± 0.05	0.15 ± 0.04	0.022
**MAP (mmHg) Preoperative**	103.12 ± 11.58	103.22 ± 11.73	0.980
**MAP (mmHg) Postoperative**	108.12 ± 9.76	103.45 ± 9.54	0.049
**SpO₂ (%) Preoperative**	97.98 ± 1.65	98.02 ± 1.63	0.942
**SpO₂ (%) Postoperative**	94.89 ± 2.11	97.45 ± 1.88	**<0.001**
**Pharyngeal pH Preoperative**	6.53 ± 2.29	6.51 ± 2.28	0.982
**Pharyngeal pH Postoperative**	4.62 ± 1.95	5.76 ± 2.05	0.029

Sixty patients with diabetic gastroparesis scheduled for surgery under general anesthesia, including 34 males and 26 females, aged 27-65 years, with an average of 48.19 ± 7.23 years were evaluated. Of these, 9 patients (15%) experienced reflux (n=6, 10%) or macro-aspiration (n=3, 5%) events, while 51 patients (85%) had no such events.

### Gastric Emptying Indices

Patients with reflux/macro-aspiration events had significantly higher CSA (3.28 ± 0.35 cm² vs. 3.07 ± 0.29 cm², t=2.122, P=0.038), GV (12.15 ± 3.28 mL vs. 10.12 ± 2.76 mL, t=2.563, P=0.013), and GV/W (0.17 ± 0.05 mL/kg vs. 0.15 ± 0.04 mL/kg, t=2.347, P=0.022) compared to those without events. No significant differences were observed in preoperative MAP (103.12 ± 11.58 mmHg vs. 103.22 ± 11.73 mmHg, t=0.025, P=0.980) or SpO2 (97.98 ± 1.65% vs. 98.02 ± 1.63%, t=0.073, P=0.942) between groups. Postoperatively, patients with reflux/macro-aspiration events had significantly higher MAP (108.12 ± 9.76 mmHg vs. 103.45 ± 9.54 mmHg, t=2.013, P=0.049) and lower SpO2 (94.89 ± 2.11% vs. 97.45 ± 1.88%, t=3.876, P<0.001) compared to those without events. No significant differences were observed in preoperative pharyngeal pH (6.53 ± 2.29 vs. 6.51 ± 2.28, t=0.023, P=0.982) or gastric protease levels (51.78 ± 13.88 ng/mL vs. 51.66 ± 13.79 ng/mL, t=0.026, P=0.979) between groups. Postoperatively, patients with reflux/macro-aspiration events had significantly lower pharyngeal pH (4.62 ± 1.95 vs. 5.76 ± 2.05, t=2.234, P=0.029) and higher gastric protease levels (62.78 ± 10.92 ng/mL vs. 55.88 ± 11.34 ng/mL, t=2.879, P=0.006) compared to those without events. As shown in Table-1.

### Predictive Role of Ultrasound

Logistic regression analysis showed that higher CSA (OR=1.45, 95% CI: 1.02-2.06, P=0.039), GV (OR=1.22, 95% CI: 1.04-1.43, P=0.015), and GV/W (OR=1.38, 95% CI: 1.03-1.85, P=0.031) were significantly associated with an increased likelihood of reflux/macro-aspiration events.

## Discussion

Diabetes is a metabolic disorder causing chronic hyperglycemia due to insulin deficiency (type 1) or resistance (type 2), with emerging evidence implicating excessive glucagon secretion [14]. Its prevalence varies by region, influenced by genetics and lifestyle, rising with urbanization [15]. Poor glycemic control leads to complications, including delayed gastric emptying (DGP), affecting up to 60% of patients and increasing aspiration risk during anesthesia [16][17]. Gastric ultrasound, using low-frequency (adults) or high-frequency (children) probes, assesses gastric volume, with fasting levels typically ≤1.5 mL/kg in healthy adults, minimizing aspiration risk [18][19].

Our provides compelling evidence for the utility of gastric antral ultrasound in assessing the risk of reflux and macro-aspiration in diabetic patients with gastroparesis undergoing surgical procedures. These findings align with existing literature, such as a scoping review by Xiao et al. (2021) [20], which highlighted the limited and contradictory data on fasting gastric content in diabetic patients, underscoring the need for reliable assessment tools like ultrasound. Similarly, a meta-analysis by Baldawi et al. (2023) reported increased antral CSA and gastric residual volume in diabetic patients, supporting the predictive value of these ultrasound measurements in identifying high-risk individuals [21].

The clinical implications of these findings are profound, as they advocate for the integration of gastric antral ultrasound into preoperative protocols to enhance patient safety in diabetic gastroparesis patients. By identifying elevated CSA, GV, and GV/W, anesthesiologists can implement tailored strategies to mitigate aspiration risks, such as prolonged fasting, pharmacological interventions like metoclopramide, or rapid sequence induction techniques. A study by Sastre et al. (2022) further emphasized the importance of ultrasound in diabetic patients with dysautonomia, reporting a higher prevalence of full stomach (22.9% in dysautonomia-positive diabetics vs. 16.1% in those without and 13.2% in healthy controls), suggesting that this subgroup may benefit most from ultrasound screening [22]. Another meta-analysis by Simadibrata et al. (2023) provided comprehensive evidence of ultrasonography’s utility, demonstrating wider antral CSA, prolonged gastric emptying times, and reduced gastric emptying rates in diabetic gastroparesis patients compared to healthy controls [23]. Additionally, a study comparing diabetic and non-diabetic older adults undergoing total knee arthroplasty found significantly higher residual gastric volume in diabetics at the second surgery (75.1 ± 43.2 mL vs. 35.9 ± 25.9 mL, P=0.002), reinforcing the need for ultrasound in this population [24].

This study found that the postoperative MAP, SpO2, pharyngeal pH in the observation group were better than those in the control group. This suggests that performing gastric antral ultrasound examination and measuring gastric contents before anesthesia, and intervening based on the measurement results, can reduce the patient's stress response and pharyngeal pH, gastric protease levels, and ensure patient safety.

This study also found that the incidence of reflux and aspiration in the observation group was significantly lower than that in the control group, suggesting that the risk of reflux and aspiration in patients undergoing surgery for diabetes gastroparesis could be reduced by carrying out ultrasonic examination of the gastric antrum and evaluating the risk of reflux and aspiration. Therefore, adjusting the surgical strategy and carrying out relevant interventions can reduce the risk of reflux and aspiration.

The reason for this is that effective risk stratification and volume control can be achieved through gastric ultrasound examination, where CSA>3.40cm ² is defined as an "unacceptable risk" and surgery can be postponed accordingly. When GV/W is less than 0.8 mL/kg, the supine gastric pressure is less than the resting pressure of the lower esophageal sphincter (normal about 15-30 mmHg), which can physically block reflux. Although conducting gastric ultrasound examination cannot directly accelerate gastric emptying, it can create an individualized decision-making system based on objective indicators, thereby accurately identifying hidden high-risk patients and avoiding blind surgery. At the same time, it can also improve the effectiveness of conventional intervention measures for inflammation, optimize the efficacy of acid suppression drugs, and avoid excessive airway manipulation. This decision-making system can effectively block the relevant links that affect reflux aspiration (gastric content volume → reflux dynamics → degree of injury), thereby reducing the risk of reflux aspiration and synchronously improving physiological stress and mucosal injury indicators, providing evidence-based support for perioperative management of DGP patients.

## Conclusion

This study confirmed that ultrasound examination of gastric antrum can accurately identify DGP patients who still have high-risk gastric retention after routine fasting, and significantly reduce the incidence of reflux and aspiration by delaying surgery until gastric emptying reaches the standard. This study found that carrying out ultrasonic examination of the gastric antrum and evaluating the risk of reflux aspiration in patients with diabetes gastroparesis undergoing surgery could reduce the risk of reflux aspiration, reduce the stress response of patients, and the level of pH and pepsin in the pharynx, better maintain the stability of vital signs, and promote postoperative recovery. It is worth promoting. However, it should be noted that this study still has limitations such as a small sample size, ineffective control of confounding factors, strong subjectivity in ultrasound examination, and lack of long-term prognosis tracking. In the future, the sample size can be further expanded by developing AI assisted CSA automatic measurement algorithms to reduce human errors, conducting research on dynamic monitoring mechanisms, and attempting to upgrade intervention strategies to improve the prevention effect of reflux aspiration, ensure patient safety, and enhance surgical outcomes. This study was supported by funding provided under Fund Number 202491021.

## Conflict of Interest

None.
